# Cost of illness studies in COVID-19: a scoping review

**DOI:** 10.1186/s12962-024-00514-7

**Published:** 2024-01-18

**Authors:** Majid Nakhaee, Masoud Khandehroo, Reza Esmaeili

**Affiliations:** 1 Social Development and Health Promotion Research Center, Gonabad University of Medical Sciences, Gonabad, Iran; 2 Department of Community Medicine, School of Medicine, Social Development and Health Promotion Research Center, Gonabad University of Medical Sciences, Gonabad, Iran; 3 Department of Public Health, School of Health, Social Development and Health Promotion Research Center, Gonabad University of Medical Sciences, Imam Khomeini Avenue, Gonabad, Khorasan 9691793718 Iran

**Keywords:** Cost of disease, Economic burden of disease, Burden of disease

## Abstract

**Background:**

Human communities suffered a vast socioeconomic burden in dealing with the pandemic of coronavirus disease 2019 (COVID-19) globally. Real-word data about these burdens can inform governments about evidence-based resource allocation and prioritization. The aim of this scoping review was to map the cost-of-illness (CoI) studies associated with COVID-19.

**Methods:**

This scoping review was conducted from January 2019 to December 2021. We searched cost-of-illness papers published in English within Web of Sciences, PubMed, Google Scholar, Scopus, Science Direct and ProQuest. For each eligible study, extracted data included country, publication year, study period, study design, epidemiological approach, costing method, cost type, cost identification, sensitivity analysis, estimated unit cost and national burden. All of the analyses were applied in Excel software.

**Results:**

2352 records were found after the search strategy application, finally 28 articles met the inclusion criteria and were included in the review. Most of the studies were done in the United States, Turkey, and China. The prevalence-based approach was the most common in the studies, and most of studies also used Hospital Information System data (HIS). There were noticeable differences in the costing methods and the cost identification. The average cost of hospitalization per patient per day ranged from 101$ in Turkey to 2,364$ in the United States. Among the studies, 82.1% estimated particularly direct medical costs, 3.6% only indirect costs, and 14.3% both direct and indirect costs.

**Conclusion:**

The economic burden of COVID-19 varies from country to country. The majority of CoI studies estimated direct medical costs associated with COVID-19 and there is a paucity of evidence for direct non-medical, indirect, and intangible costs, which we recommend for future studies. To create homogeneity in CoI studies, we suggest researchers follow a conceptual framework and critical appraisal checklist of cost-of-illness (CoI) studies.

## Introduction

Cost-of-illness (CoI) and burden of disease (BoD) studies are valuable complementary tools to evaluate the burden of a condition. BoD deals with the mortalities and disabilities that are attributable to a specific condition in terms of DALY (YLD + YLL) as well CoI studies try to measure the economic burden of an illness [1, 2].

Tarricone claims that “CoI have an important role in health economics as a decision-making tool” [3]. CoI studies can provide baseline and relative values about the economic consequences of diseases and also estimate the saved resources due to preventive plans [1]. In other words, the opportunity cost of the decision will be revealed which helps in choosing wisely. So, regarding the scarce resources for developing health systems, CoI findings are essential for further analysis of priority setting, resource allocation, economic evaluation and health technology assessment in health systems.

**Fig. 1:**
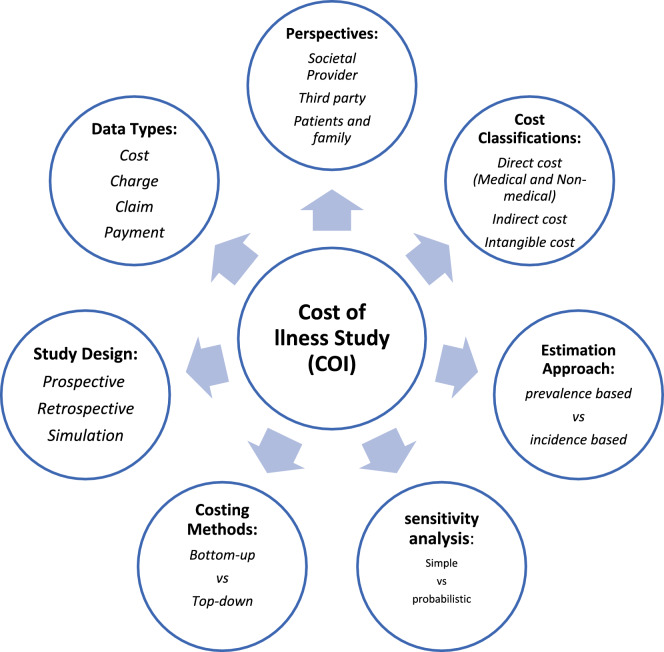
Conceptual framework of cost-of-illness studies components

CoI studies have a wide set of characteristics that form their methodologies and potential generalizability. In this setting, we should select our study design, perspective, costing methods, cost categories and et cetera [3, 4]. A conceptual framework of CoI characteristics is shown in Fig. 1. An appropriate conceptual framework would reduce methodological heterogenicity.

There is a growing body of literature dealing with CoI, and also some systematic reviews have been conducted to describe the CoI studies done for different conditions or even in a specific geographic area. For example, García-Pérez (2016) overviewed COI studies carried out about 42 rare diseases [5], and Brodszky et al. published a multi-country review of COI studies from the central and eastern European countries [6]. In their reviews, heterogeneities of CoI studies were concluded as a main issue.

COVID-19 imposed a considerable socioeconomic burden on governments, health systems and patients around the world [7, 8]. The stewards of the health systems will not address this condition unless researchers provide real-world evidence. Without a real understanding of what is happening, priority- setting exercises in the health system will be imperfect. Although some CoI studies have targeted COVID-19 in different countries, a valid estimation of associated financial burden and opportunity cost strictly depends on how CoI study components were applied. An overview of CoI studies components associated with different health conditions is beneficial to identify common limitations, strengthen methodological issues and avert misleading. This scoping review aimed to map the cost-of-illness studies associated with COVID-19.

## Methods

This scoping review was based on the original articles associated with the cost of COVID-19, published from 2019 to 2021. For collecting data, a systematic search was applied in databases including PubMed, Web of Science, ProQuest, Google Scholar, Scopus, and Science Direct. The keywords used in this search included cost, cost-of-illness, costing, economic burden, COVID-19, covid, corona, coronavirus, and other similar words that were designed with appropriate combinations by the instructions defined in each search engine. In addition, a manual review was done of the selected studies’ reference lists. Inclusion criteria were the cost of illness studies conducted during the Covid-19 epidemic and published in English. While the first cases of COVID-19 were confirmed in December 2019, we decided to review published articles until the end of 2021. Letters to the editor, reports, and articles published in conferences and qualitative studies were excluded.

To find related articles, three authors evaluated retrieved records separately and also in joint meetings. In this way, in the first stage, the titles of the articles were screened, and irrelevant records were excluded. Then the abstract and the full text of the included articles were reviewed, and unrelated studies were discarded.

**Fig. 2:**
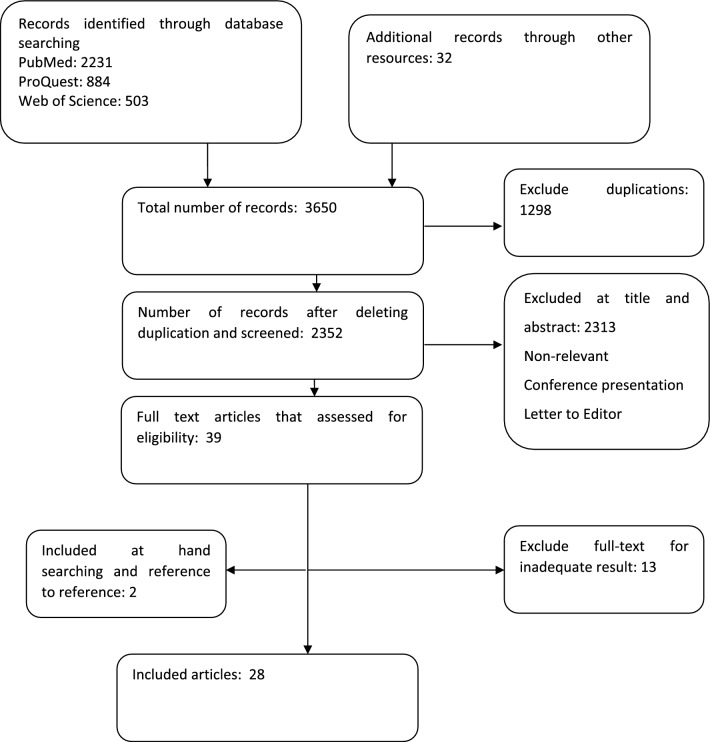
PRISMA flow chart of search

Endnote X7 resource management software was used to organize, sort, and identify duplicates. The required information was extracted from the selected articles in a pre-designed data extraction form (Fig. 2).

## Result

2352 records were found after the search strategy application. After an initial screening of titles and abstracts, 2313 were excluded. Then 39 full-text articles were retrieved and assessed for eligibility. Finally 28 articles were retained for inclusion in this scoping review. The majority of studies tried to estimate a unit cost measure such as cost per patient, per admission or per day. The average cost of hospitalization per patient per day ranged from 101$ in Turkey to 2,364$ in the United States. 42.8% of studies attempted to estimate the national burden of COVID-19 in term of total expenditure, percent of country GDP or per 1000 population. More detailed extracted data is available in Table 1.

Table 1 shows the main characteristics of the COI studies. The retrieved studies originated around worldwide including: China [9–11], Turkey [12–15], Kenya [16, 17], United States [18–21], Iran [22, 23], South Africa [24, 25], Saudi Arabia [26], Germany [27], Greece [28, 29], Brazil [30, 31], Europe [32], Colombia [33], Ghana [34], India [35], and South Korea [36]. The retrieved studies’ time period differed from only two months [11, 13, 34] to 12 months [15]. As can be seen from Table 2, most studies (71.4%) adopted a prevalence-based approach. Only five studies (17.9%) adopted an incidence-based estimation approach with a patient’s lifetime horizon. About half of the studies (46.4%) adopted the health system as the study perspective and 35.7% chose the payer perspective (including: MEDICARE, insurance companies);and only one study used the patient perspective. However, the perspective was not always explicitly stated. 24 (85.7%) Of the 28 studies attempted to estimate the costs using bottom-up costing method. Only one study applied top-down costing, and one used a mixed costing approach. The majority of studies (82.1%) measured only direct medical costs, and one study tried to estimate indirect costs. 17.9% of studies measured mixed cost types (3.6% both direct medical and non-medical, 7.1% both direct medical and indirect, and 3.6% both direct and Indirect). The strategies to identify resource utilization were varied; 10 studies (35.7%) used charge as a proxy.

The predominant study design for data collection was a retrospective cross-sectional design, followed by cohort design studies. Only 21.4% of studies applied sensitivity analysis.

**Table 1:** Summary of cost-of-illness studies in COVID_19

Author (year)	Country	Year of study	Unit cost (AIC)	National burden	Ref.
Ahmad Faramarzi (2021)	Iran	Between Feb 19, and Sep 21, 2020	$671.4 per patient	Total costs due to absenteeism were estimated to be nearly $1.3 million, with an average of $671.4 per patient	[22]
Alvis-Zakzuk N (2021)	Colombia	From March 15 and May 29, 2020	$1688 per patient	–	[33]
Anna Miethke-Morais (2021)	Brazil	Between March 30 and June 30, 2020	US$12,637.42 per admission US$919.24 per day	The total cost of the hospitalizations was US$41,122,173.39	[30]
Athanasakis (2020)	Greece		443.1 and 2245.5 euros per day in the general ward and ICU respectively	6.53 million international dollars per 1000 confirmed	[28]
Bartsch (2020)	United States		$3,045 per patient	If 80% were to get infected, $654.0 billion in direct medical costs and If 20% were to get infected $163.4 billion in direct medical costs	[18]
Cenk Teker (2021)	Turkey	1 year (04.2020–04.2021)	Per-day unit cost for an inpatient is estimated to be 1.184,63 TRY and ICU 15.466 TRY	–	[15]
Cleary (2021)	South Africa	Up to the end of May 2020	75,127 ZAR per admission	–	[24]
Czernichow (2020)	Europe	The first 6 months of 2020	Between 15,831 EUR to 30,982 EUR based on BMI	Total direct costs of care for COVID-19 in Europe EUR: 13.9 billion	[32]
Dr. Edwine Barasa and Dr. Angela Kairu (2020)	Kenya		Per patient costs in ranging from KES 21,359 per day to KES51,684		[16]
Edwine Barasa (2021)	Kenya		From 18.89 to 599.51 $ based on disease condition	–	[17]
EKİNGEN, Erhan (2021)	Turkey	Discharged in June 2020	The average invoice amount was $653.03 per patient	–	[12]
Ergun Oksuz (2021)	TURKEY	March 11 and July 31, 2020	PPP $5557.9 ± 7473.4 per patient	The direct annual medical cost of COVID-19 was estimated at PPP$ 2.1 billion and PPP$ 1.9 billion for inpatient and outpatient. direct medical burden corresponds to 2.0% of the government health expenditures and 0.8 per GDP	[14]
Ghaffari Darab (2021)	Iran	Between March and July 2020	The direct medical cost was $ 3755 per person Indirect estimated $ 11,634 per person	Economic burden of the disease in the country for inpatient cases with the definitive diagnosis was $ 1,439,083,784 in 2018	[23]
Habip Gedik (2020)	Turkey	Between 17 March and 11 May 2020	$ 881.75 per clinical patient. The mean cost per ICUP was $2924	–	[13]
Hamza Ismaila (2021)	Ghana	August and September 2020	US$11,925 per patient	–	[34]
Hebert Luan Pereira Campos dos Santos (2021)	Brazil	February and December 2020	4864 $ per hospitalization	Total expenditure R$ 2.2 billion	[31]
Huajie Jin (2020)	China	During 1 January–31 March 2020	US$ 3192.76 per patient	The total societal cost was US$ 383.02 billion (2.7% of China’s GDP	[10]
Ijeoma Edoka (2021)	South Africa		The total average cost per patient per day ranged from 62 to 830 based on the ward, need for invasive, need for supplemental oxygen	–	[25]
Jeck (2021)	German	From January 01, 2020 to Sep 30, 2020	Median treatment costs from EUR 900 to EUR 53,000 per patient	–	[27]
Kamini N Reddy (2021)	India	From June 2020 to December 2020	The median and mean direct medical cost of hospitalization was 2742.91 and $ 3192.06 respectively	–	[35]
khan (2020)	Saudi Arabia	1 March and 29 May 2020	12,912$ per patient		[26]
Maltezou (2021)	Greece	From 26 Feb 2020 to 3 May 2020	–	Total cost for the management of health care personnel during the first epidemic in Greece 1.735.830 Euros Overall, indirect costs for health care personnel were 1,409,720 Euros(81.2% of total costs)	[29]
Manuela Di Fusco (2021)	United States	From 1 April to 31 October 2020	–	–	[19]
Moran Dong (2021)	China	From January 15 to April 27, 2020	The median of total hospitalization costs of COVID-19 cases was $2,869.4 per patient	–	[9]
Ohsfeldt (2021)	United States	April 1 to December 31, 2020	–	–	[20]
Su Yeon Jang (2021)	Korea	As of May 15, 2020	The average cost of medical expenses was USD 1193.7	–	[36]
Xue-Zheng Li (2020)	China	Between 24 January and 16 March 2020	USD 6827 per treated episode	Around USD 0.49 billion were expected for clinical management of COVID-19 (about 0.2% of China healthcare expenditure in 2019)	[11]
Yuping Tsai (2021)	United States	During 1 April through 31 December 2020	The mean hospitalization was $21,752 per patient and The mean cost per outpatient visit was $164	Total medical costs for COVID-19 related medical care for patients were $6.3 billion	[21]

**Table 2:** Components of cost-of-illness studies in COVID_19

Cost of illness study components		Frequency ( %)	Case study
Epidemiological spproach	Prevalence based	20 (71.4)	[9–15, 19–22, 26, 27, 29–33, 35, 36]
	Incident based	5 (17.9)	[16, 18, 23, 25, 34]
	Not mentioned	3 (10.7)	[17, 24, 28]
Study design for data collection	Prospective	2 (7.1)	[15, 30]
	Retrospective (including protocol-based study)	25 (89.3)	[9–14, 16, 17, 19–29, 31–36]
	Scenario-based simulation	1 (3.6)	[18]
Study perspective	Health system	13 (46.4)	[9, 15–20, 22, 24, 25, 28, 30, 34]
	Payer	10 (35.7)	[11–14, 21, 26, 27, 29, 33, 36]
	Government	2 (7.1)	[31, 35]
	Patient	1 (3.6)	[23]
	More than one perspective	1 (3.6)	[10]
	Not mentioned	1 (3.6)	[32]
Costing method	Bottom-up	24 (85.7)	[9–27, 29, 33–36]
	Top-down	1 (3.6)	[31]
	Both	1 (3.6)	[30]
	Not mentioned	2 (7.1)	[28, 32]
Cost type	Direct medical cost	23 (82.1)	[9, 11–14, 16–21, 24–28, 30–36]
	Direct non-medical cost	0 (0)	–
	Indirect medical cost	1 (3.6)	[22]
	More than one cost type	4 (14.3)	[10, 15, 23, 29]
Cost identification	Cost	2 (7.1)	[15, 22]
	Charge	10 (35.7)	[9, 16, 18, 20, 24, 26–28, 33, 34]
	Expenditure	1 (3.6)	[31]
	Claim	4 (14.3)	[12, 14, 21, 36]
	More than one cost identification	10 (35.7)	[10, 11, 13, 17, 19, 23, 25, 29, 30, 35]
	Not mentioned	1 (3.6)	[32]
Sensitivity analysis	Done	6 (21.4)	[10, 17–19, 24, 25]
	Not stated	22 (78.6)	[9, 11–16, 20–23, 26–36]

## Discussion

This scoping review aimed to map the cost-of-illness studies associated with COVID-19. We found that over a couple of years since the beginning of the COVID-19 pandemic, a considerable number of health economics literature explored the associated cost of COVID-19 around the world. Similar to CoI studies in other conditions [5], most studies have adopted a prevalence-based approach. The prevalence approach estimates the economic burden of a diseases for a specific period, but in incidence based approaches cost are estimated for life-time design that are followed over the course of a condition until recovery or death. So prevalence-based approach is the most suitable applied design for COVID-19.Tarricone [3] recommended the prevalence approach for estimating the global burden of a condition, identifying its cost components, and finally helping in the planning of cost-containment policies.

Most of the articles had benefited from real world cost through retrospective data collection design. Also, one article followed Monte Carlo simulation design [18]. Takemoto et al. reported various methods to measure the cost rotavirus disease in Latin America and the Caribbean while most of them were based on retrospective administrative database analysis [37].

The perspective of CoI studies determines which cost items could be included in the analysis. The health system perspective was the most common for COVID-19. To address the COVID-19 crisis, governments allocated compensatory budgets and subsidies to health systems and healthcare providers beyond the formal bills for payers and patients. Thus, the health system’s perspective would depict a proper estimation of the economic burden of COVID-19. GarcíaPérez et al. [5], reviewed the CoI studies associated with rare diseases, in which societal perspective was the most common in that condition. Also in a systematic review conducted by Oliveira et al., they showed that 55% of studies adopted the society’s perspective, while 45% used the perspective of the public health service provider or a private budget holder [38]. Societal perspective include total cost is incurred by all society’s financing agents (people, governments, insurance companies, …) but other perspectives cover a particular part of mentioned total cost. So, government and health system perspectives can be different based on unique health stewardship in any country.

Only one study has used the top-down costing method, others have used the bottom-up method. However, some studies have not explicitly stated the costing method. One reason may be that initial data on disease costs—especially on Covid-19 disease—are available through patient records or HIS, while access to macro-level cost information is difficult for researchers. The methodological differences between top-down and bottom-up costing methods could make it difficult to compare and synthase the retrieved findings. In a similar finding obtained by Strilciuc et al., it was found that about 31% of the studies used the bottom-up costing method in estimating costs, only 4% used the top-down method, and in 28% of the studies, the costing method used was not explicitly mentioned [39].

More than 35% of studies have included only charges as a proxy for resource utilization. The main reason may be the accessibility of the data through hospital records. The rest of the studies have used other kinds of cost identifications such as claim (14.3%), expenditure (3.6%), and cost (7.1%), however some of them have not explicitly stated. Although these share percent are according to reported methods but stated cost maybe not appropriate with the applied cost data.

### Study strengths and limitations

To our best knowledge, this is the first scoping review study conducted about the cost-of-illness of COVID-19. We tried to find all the published associated studies by searching in several search engines and available sources; we also used multiple and specific search terms in order to increase search sensitivity. The principal limitation of this review was the variance in the design of retrieved records considering most of them did not follow the same conceptual framework and/or a critical appraisal checklist of cost-of-illness (CoI) studies. This limitation created difficulties for extracting targeted data. So we conducted some group discussion and communicated with the authors to make common sense.

## Conclusion

Although COVID-19 imposed a huge economic burden on health systems around the world, this study revealed a wide heterogeneity among COI studies focused on COVID-19. The majority of CoI studies estimated direct medical costs and there is a lack of evidence for direct non-medical, indirect and intangible costs, which we recommend for future studies.

Different concepts for “cost identification” were used such as charge, cost, payment, claim, and expenditure which are used instead of each other in CoI studies, whereas each of them implies different values in health care costing. Only a few CoI studies have tried to estimate the economic burden of Covid-19 at a national level, so the comparison between countries and global estimation isdifficult to apply. We suggest researchers follow a conceptual framework and critical appraisal checklist of cost-of-illness (COI) studies.

## Data Availability

Not applicable.
